# Autologous Rib Cartilage Reconstruction After Silicone Implant Removal in a Patient With Bilateral Cleft Lip and Palate: A Case Report

**DOI:** 10.7759/cureus.58452

**Published:** 2024-04-17

**Authors:** Taiki Nagatsuka, Naoki Matsuura, Edward H Ntege, Yusuke Shimizu

**Affiliations:** 1 Plastic and Reconstructive Surgery, University of the Ryukyus Hospital, Nishihara City, JPN

**Keywords:** septal extension graft, silicone implant, rib cartilage, rhinoplasty, cleft lip nose deformity

## Abstract

Cleft lip rhinoplasty (CLR) corrects nasal deformities in cleft lip and palate patients. However, limitations exist in some countries like Japan regarding the use of silicone implants for CLR. While historical reports mention their use since the 1980s, long-term data is lacking. This case report describes a 53-year-old Japanese woman with bilateral cleft lip and palate who received a CLR with a silicone implant over 30 years ago. The implant calcified, causing nasal dorsum skin hardening and thinning, raising concerns of extrusion. To prevent potential extrusion, the implant was removed and replaced with autologous seventh rib cartilage grafts. Various grafting techniques were used for basal support, dorsal augmentation, and nasal tip refinement. The postoperative evaluation showed excellent results with no complications. This case highlights the importance of long-term follow-up after CLR with silicone implants and advocates for autologous rib cartilage as a reliable alternative. Reporting such cases is crucial for informing patient management and research on the long-term safety of silicone implants in CLR.

## Introduction

Cleft lip and palate are among the most common cranio-maxillofacial congenital anomalies, comprising 65% of all head and neck deformities and affecting approximately 1 in 600 to 800 live births (about 1.7 in 1000) globally [1,2]. In Japan, the prevalence rate of cleft lip and palate is approximately 1.46 in 1000 [3]. The management of cleft lip and palate is complex and necessitates a multidisciplinary approach, with surgical correction being a crucial component. Rhinoplasty plays a significant role in the surgical management of cleft lip and palate, as patients often present with associated nasal deformities [2].

In Japan, various surgical materials, including nasal septum cartilage, auricular cartilage, and rib cartilage, have been extensively utilized for cleft lip rhinoplasty (CLR) [4-6]. Although Namba et al. documented the use of silicone implants during CLR in 1985, further reports of such cases in the field of plastic surgery in Japan remain scarce [7]. The lack of approval from the National Health Insurance system has deterred surgeons from routinely employing silicone implants [8]. Nonetheless, some surgeons have opted for these implants in reconstructive surgery, especially when patients with cleft lip seek aesthetic enhancement of the nose.

Despite offering potential short-term cosmetic benefits, silicone implants carry the risk of late complications, including skin thinning, exposure, calcification, and breakage, which may manifest several decades after implantation [9]. In this report, we present the case of a patient with bilateral cleft lip and palate who underwent CLR with a silicone implant over 30 years ago. Over time, the implant calcified, leading to skin hardening and thinning of the nasal dorsum. To prevent implant exposure, removal, and reconstruction were performed, yielding highly satisfactory results. This case underscores the necessity of long-term monitoring for patients with silicone implants and emphasizes the pivotal role of plastic surgeons in managing these complications.

## Case presentation

A 53-year-old Japanese woman presented to the Department of Plastic and Reconstructive Surgery at the University of the Ryukyus Hospital in October 2022 with a 10-day history of a persistent ridge on her nasal dorsum. Referred by a physician for further evaluation and management, the patient reported a history of complete bilateral cleft lip and palate with approximately six prior surgeries since childhood, details of which were limited. The most recent procedure, performed around age 19, involved CLR with implant insertion. The patient reported no prior complications or concerns related to her nose since undergoing CLR with implant insertion 30 years ago. Furthermore, she denied any history of factors typically associated with nasal dorsum ridge formation or implant complications such as trauma, infection, or inflammation. The patient’s medical and family history were unremarkable.

**Figure 1:**
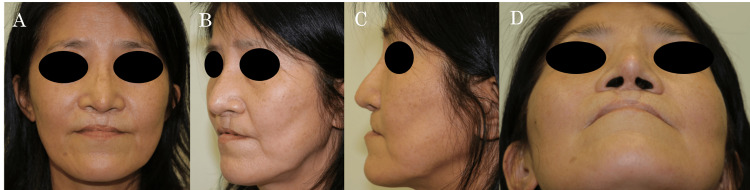
Preoperative photographs at initial consultation (A) Frontal view, (B) Left oblique view, (C) Left lateral view, and (D) Worm's eye view demonstrating thinned skin on the nasal dorsum with mild redness

General examination revealed a healthy patient with no pallor, edema, jaundice, or cervical/generalized lymphadenopathy. Systemic findings were unremarkable. Local examination identified a palpable silicone implant with surrounding capsules on the nasal dorsum. The overlying skin appeared thin and mildly edematous (Figures 1A-1D).

**Figure 2:**
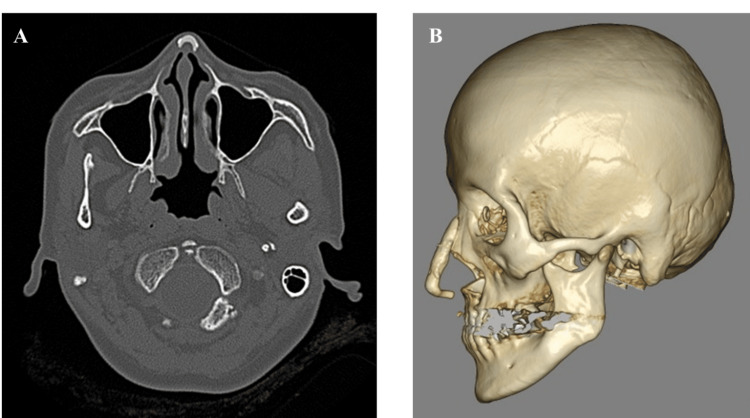
Preoperative CT scan images (A) Coronal section and (B) 3D reconstruction revealing an L-shaped silicone implant extending from the nasal dorsum to the base of the columella

Computed tomography (CT) scan demonstrated an L-shaped silicone implant (long axis: length, 48 mm; width, 7 mm; short axis: length, 19 mm; width, 4 mm) extending from the nasal dorsum to the columellar base (Figure 2). Routine hematological tests were within normal limits.

The treatment plan consisted of implant removal with subsequent rhinoplasty using rib cartilage grafts for nasal septal lengthening, tip augmentation, and dorsal augmentation. As established guidelines are lacking for managing this rare condition, the treatment plan was tailored to address the unique challenges posed by the patient's prior surgery and the need for implant removal and reconstruction. Well-established techniques from both CLR and cosmetic rhinoplasty were combined to create a comprehensive surgical approach optimized for the patient's needs [4,10].

**Figure 3:**
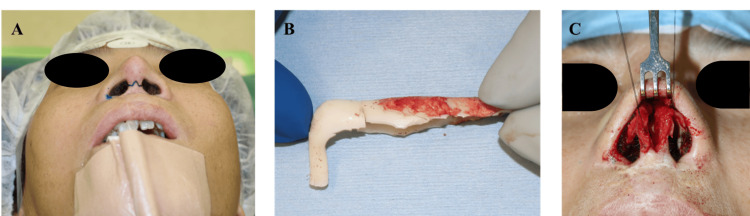
Intraoperative photographs (A) Planned incision lines on the skin, (B) Removed silicone implant, (C) Bilaterally displaced lower lateral cartilage

An inverted V-shaped incision provided access to the nasal bridge, followed by a subcartilaginous incision to access the nasal cavity (Figures 3A, 3B). Significant implant calcification presented a challenge during removal (Figure 3C). However, we were able to preserve most of the capsule. This helped maintain the structural integrity of the overlying thinned skin, reducing the risk of perforation or excessive weakness.

**Figure 4:**
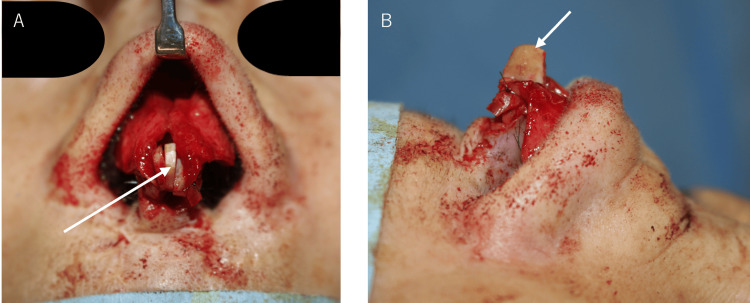
Intraoperative photographs (A) Frontal view and (B) Left lateral view showing the placement of spreader graft (right side), batten graft (left side), and end-to-end graft (white arrow) on the nasal septum.

A 4-cm segment of the right seventh rib cartilage was harvested with its perichondrium and anterior rectus abdominis from a right anterior thoracic incision. The rib cartilage was then divided using the oblique split method for graft preparation [4]. Extended septal grafts were positioned on both the left (batten type) and right (spreader type) sides of the nasal septum cartilage. An end-to-end extended septal graft was secured between the two (Figures 4A, 4B). This extended graft was then fixed to the medial and middle crura of the alar cartilage.

**Figure 5:**
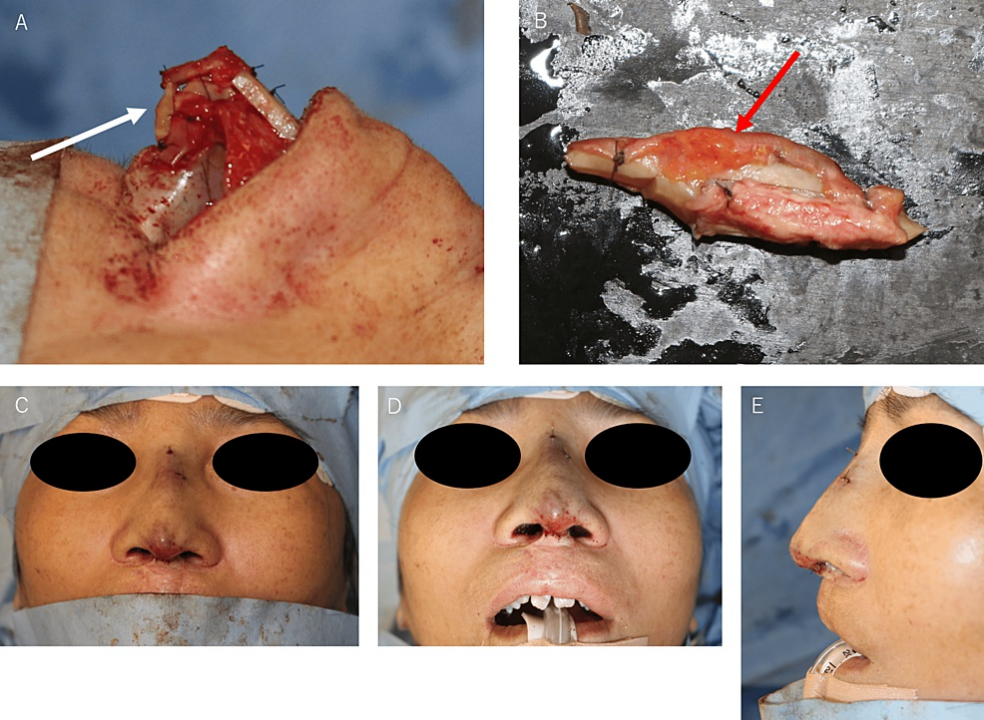
Intraoperative photographs (A) Placement of a columellar strut (white arrow) between the medial crura, a shield graft at the nasal tip, and a derotation graft on the cephalic side, (B) Dorsal onlay graft constructed from two layers of rib cartilage with rectus abdominis fascia (Red arrow) on the skin side and rib cartilage perichondrium on the nasal bone side, (C) Frontal view, (D) Worm's eye view, and (E) Left lateral view after closure, showing temporary congestion of the nasal tip skin, which gradually subsided.

A columellar strut was placed between the medial crura for shaping and stabilizing the nasal apex. A shield graft was secured at the nasal apex, followed by a derotation graft on the cephalic side (Figure 5A). For dorsal augmentation, two layers of rib cartilage were positioned on the nasal dorsum, under the preserved capsule. The deep layer was placed against the nasal bone while the superficial layer was oriented with the perichondrium facing the bone side and the anterior rectus abdominis fascia facing the skin (Figure 5B). The anterior rectus abdominis fascia served as a barrier between the cartilage graft and the preserved capsule on the skin side, aiming to provide a smooth contour and minimize the risk of graft visibility or palpability. Diced rib cartilage fragments were used to address any remaining nasal dorsum irregularities. Finally, the nasal column and cavity were carefully sutured (Figures 5C-5E).

**Figure 6:**
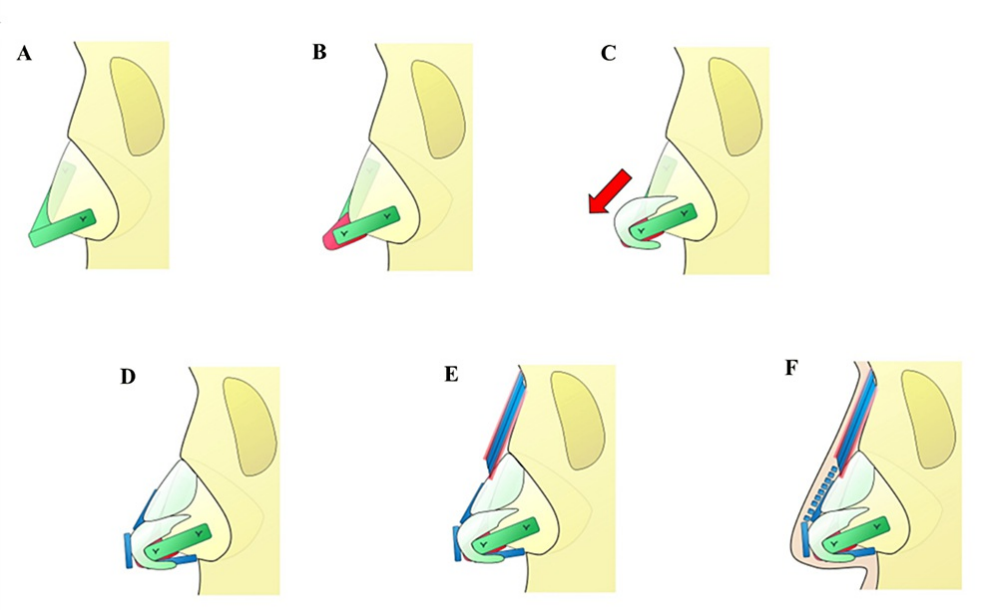
Schematic representation of nasal graft placement (A) Batten graft (green) positioned on the left side and spreader graft (green) positioned on the right side of the nasal septum, (B) End-to-end septal extension graft (red) positioned and secured between the batten graft and spreader graft, (C) Alar cartilage extended and secured along with the septal extension graft, resulting in anterior lengthening and downward rotation to prevent an upturned nasal deformity, (D) Columellar strut (blue) secured between the medial crura, Shield graft (blue) positioned at the nasal tip, and derotation graft (blue) positioned on the cephalic aspect to reinforce the downward rotation of the alar cartilage, (E) Dorsal onlay graft (blue) composed of two layers of rib cartilage with a superficial layer of rectus abdominis fascia (pink) and a deep layer of cartilage perichondrium (pink), secured to the nasal bone, (F) Crushed rib cartilage (blue) layered from the nasal dorsum to the nasal tip to create a smooth nasal contour

A detailed depiction of the procedure is provided in the accompanying schematic representation (Figure 6).

**Figure 7:**
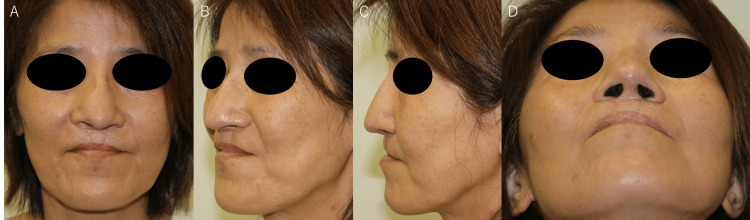
Postoperative photographs at 11 months (A) Frontal view, (B) Left oblique view, (C) Left lateral view, and (D) Worm's eye view demonstrating a well-maintained external nasal morphology comparable to the preoperative state

Postoperatively, no graft deviation or infection was observed. At the one-year follow-up, the patient's external nose had a natural appearance, and she expressed high satisfaction with the outcome (Figures 7A-7D).

## Discussion

In Japan, nasal septum cartilage, auricular cartilage, and rib cartilage have been widely adopted as surgical materials for rhinoplasty in patients with cleft lip [5,6,9]. Although a study published in 1985 reported the use of silicone implants during rhinoplasty for cleft lip, there have been no such reports in the field of plastic surgery in Japan since then [7]. Silicone implants are not approved by the National Health Insurance system, which has likely discouraged their use by surgeons. Nevertheless, some surgeons have used these implants for plastic reconstructive surgery, and patients with cleft lip have sought silicone implants at cosmetic surgery clinics to achieve a more aesthetically pleasing external nasal shape. Consistent with previous reports, the precise number of such cases is unknown but is estimated to be considerable [7].

In recent years, rhinoplasty with silicone implants has been performed for cleft lip deformities in other Asian countries such as Korea and Vietnam [10]. Some studies have reported no significant differences in adverse events between silicone implants and autologous grafts [11]. Despite the lack of insurance coverage, the use of silicone implants during rhinoplasty for cleft lip deformities can be a simple technique that may provide good short-term results in Japan, given the similarity in skin characteristics between Japanese and other Asian populations.

However, silicone implants can cause late complications, such as skin thinning and nasal capsular contracture, including skin hardening, color change, dislocation, extrusion, and breakage, long after implantation [12,13]. Additionally, a study by Kim et al. found that silicone implants in Asian rhinoplasty can lead to long-term complications, including implant displacement, calcification, and infection [14]. These findings underscore the importance of long-term follow-up for patients with silicone implants.

Our patient did not experience major problems for more than 30 years after implant insertion and was satisfied with the cosmetic outcome. However, calcification of the implant and thinning of the skin likely occurred gradually over time. Regular follow-up by a plastic surgeon could have detected these changes earlier, regardless of the patient's awareness. Calcification of silicone implants increases the risk of complications and may necessitate implant removal [12]. Therefore, long-term follow-up after silicone implant insertion is essential. Ideally, follow-up should be performed at the facility where the implant was inserted; however, this may be impractical in many cases. We believe there is an unmet need for plastic surgeons who can evaluate such patients and recommend appropriate implant removal and reconstruction when necessary.

For our patient, we performed nasal septum extension, nasal tip augmentation, and dorsal augmentation using rib cartilage for reconstruction after implant removal, achieving a satisfactory aesthetic outcome. Patients often have high expectations for implant removal and reconstruction. Various reconstruction methods have been proposed for silicone implant removal in aesthetic surgery [15]; however, reconstruction in patients with cleft lip is expected to be more challenging due to abnormalities in the original nasal cartilage morphology.

In the present case, removing the implant revealed outward deviation of the lower lateral cartilage, a common finding in bilateral cleft lip [16]. This outward movement likely resulted from pressure exerted by the implant widening the previously placed interdomal sutures. Successful reconstruction requires both restoring volume and establishing a stable foundation. A spreader-batten composite nasal septal extension graft offers a solution by distributing forces on the extended nasal septum in two directions [17]. This approach minimizes the risk of postoperative deviation or collapse, especially beneficial in cases with significant septal deviation and weakened nasal framework. The spreader component, positioned strategically on the concave side of the septum, aids in straightening and maintaining its corrected position. The batten component, placed on the convex side, provides additional support and stabilizes the weakened cartilage. This composite graft design disperses forces applied to the extended septal graft, enhancing overall reconstruction stability and long-term success. Additionally, the columellar strut used for nasal apex formation strengthens the caudal aspect of the greater alar cartilage [18]. The strategic placement of shield, derotation, dorsal, and diced grafts effectively re-creates the desired nasal projection from the dorsum to the tip. This versatile reconstruction method may be particularly useful for patients with cleft lip who require silicone implant removal.

Recent studies have reported favorable outcomes with the use of rib cartilage grafts in cleft lip and palate rhinoplasty. Tiong et al. found that rib cartilage grafts provided reliable structural support and satisfactory aesthetic results in patients with severe cleft lip nasal deformities [19]. Similarly, Ujam et al. demonstrated that rib cartilage grafts were effective in correcting complex cleft lip nasal deformities, with stable long-term results [20]. These findings support the use of rib cartilage grafts as a viable option for reconstruction after silicone implant removal in patients with cleft lip.

We believe that some patients with cleft lip who have received silicone implants may experience similar complications. Therefore, it is necessary to inform the public about the potential long-term consequences of silicone implants and the available treatment options. Plastic surgeons play a central role in the care of patients with cleft lip and should be prepared to appropriately manage cases involving silicone implant complications. Although the prevalence of such cases in Japan is unknown, collecting data from these patients is essential for developing optimal reconstruction methods and determining the appropriate timing of implant removal.

## Conclusions

This case report presented the development of long-term complications (implant calcification, nasal dorsum skin hardening, and thinning) in a 53-year-old Japanese woman with a history of silicone implant rhinoplasty for bilateral cleft lip and palate. Surgical management involved the removal of the silicone implant and reconstruction using autologous rib cartilage grafts for nasal septal extension, tip augmentation, and dorsal augmentation. The patient achieved a satisfactory aesthetic outcome with no postoperative complications at the one-year follow-up. The case underscores the importance of long-term follow-up, prompt intervention for implant complications, and the effectiveness of autologous rib cartilage grafts in nasal framework reconstruction after silicone implant removal in CLR. Regular monitoring, timely intervention, and a comprehensive, patient-tailored approach are crucial for optimal outcomes in such patients.
